# Barriers and Facilitators for Implementing Motivational Interviewing as a Return to Work Intervention in a Norwegian Social Insurance Setting: A Mixed Methods Process Evaluation

**DOI:** 10.1007/s10926-021-09964-9

**Published:** 2021-03-24

**Authors:** Vegard Stolsmo Foldal, Marit Solbjør, Martin Inge Standal, Egil Andreas Fors, Roger Hagen, Gunnhild Bagøien, Roar Johnsen, Karen Walseth Hara, Heidi Fossen, Ida Løchting, Hedda Eik, Margreth Grotle, Lene Aasdahl

**Affiliations:** 1 Department of Public Health and Nursing, Faculty of Medicine and Health Science, Norwegian University of Science and Technology (NTNU), Trondheim, Norway; 2 Department of Psychology, Faculty of Social and Educational Sciences, Norwegian University of Science and Technology (NTNU), Trondheim, Norway; 3 Department of Public Health and Nursing, General Practice Research Unit, Faculty of Medicine and Health Sciences, Norwegian University of Science and Technology (NTNU), Trondheim, Norway; 4 Department of Psychology, University of Oslo, Oslo, Norway; 5 Research Institute, Modum Bad, Oslo, Norway; 6 Division of Psychiatry, Tiller Community Mental Health Centre, St. Olavs Hospital, Trondheim University Hospital, Trondheim, Norway; 7 The Norwegian Labour and Welfare Service of Trøndelag, Trondheim, Norway; 8 Norwegian Advisory Unit on Complex Symptom Disorders, St. Olavs Hospital, Trondheim University Hospital, Trondheim, Norway; 9 Faculty of Health Science, Department of Physiotherapy, Oslo Metropolitan University, Oslo, Norway; 10 Research and Communication Unit for Musculoskeletal Health (FORMI) Clinic for Surgery and Neurology, Oslo University Hospital, Oslo, Norway; 11 Unicare Helsefort Rehabilitation Centre, Rissa, Norway

**Keywords:** Motivational interviewing, Return to work, Sick leave, RE-AIM, Process evaluation, Mixed-methods, Social insurance

## Abstract

*Purpose* The aim of this study was to evaluate potential barriers and facilitators for implementing motivational interviewing (MI) as a return to work (RTW) intervention in a Norwegian social insurance setting. *Methods* A mixed-methods process evaluation was conducted alongside a randomized controlled trial involving MI sessions delivered by social insurance caseworkers. The study was guided by the Reach, Effectiveness, Adoption, Implementation, and Maintenance framework using focus groups with the caseworkers. MI fidelity was evaluated through audio-recordings of MI sessions and questionnaires to sick-listed participants. *Results* Lack of co-worker and managerial support, time and place for practicing to further develop MI skills, and a high workload made the MI intervention challenging for the caseworkers. The MI method was experienced as useful, but difficult to master. MI fidelity results showed technical global scores over the threshold for “beginning proficiency” whereas the relational global score was under the threshold. The sick-listed workers reported being satisfied with the MI sessions. *Conclusions* Despite caseworker motivation for learning and using MI in early follow-up sessions, MI was hard to master and use in practice. Several barriers and facilitators were identified; these should be addressed before implementing MI in a social insurance setting.

*Trial registration* ClinicalTrials.gov: NCT03212118 (registered July 11, 2017).

## Introduction

Long-term sickness absence is a challenge for industrialized countries [1]. For the individual, prolonged sick leave is associated with adverse health outcomes, multimorbidity, increased risk of disability pension and a risk for exclusion from the labour market and economic instability [2–5]. Compared to other OECD countries, Norway has the highest sick leave [6] with a current sickness absence rate of 5.9% [7]. The leading causes for sick leave in Norway are musculoskeletal (30%) and mental health disorders (27%) [7].

In order to facilitate return to work (RTW) for sick-listed workers, early interventions have been advocated [8, 9]. Despite various targeted efforts to increase RTW, there are sparse conclusive results on what is an effective RTW approach [10–12]. To be able to better interpret the results of randomized controlled trials (RCT) and improve implementation, increased attention is given to evaluating the implementation of interventions [13, 14]. A frequently used framework for process evaluations is the Reach, Effectiveness, Adoption, Implementation and Maintenance (RE-AIM) framework [15, 16]. RE-AIM is comprised of five parts to evaluate interventions by individual factors, such as reach and effectiveness (RE), and multilevel organizational factors, such as adoption, implementation, and maintenance (AIM) of the intervention [15]. Previous studies using the RE-AIM framework to evaluate RTW interventions have emphasized the importance of evaluating potential barriers and facilitators of implementation for practice and policymakers [17, 18]. Qualitative methods are suited to explore how individuals construct meaning from their experiences, but also to investigate practices, change and processes within organizations [19]. It is recommended to use qualitative methods within the RE-AIM framework to increase the transferability of research findings to practice [20].

In an ongoing trial we are evaluating the effect of motivational interviewing (MI) on RTW, when MI is given by caseworkers at the Norwegian Labour and Welfare Administration (NAV) [21]. MI is a client-centered and directive counseling method aimed at facilitating intentional behavioral change. The method has a relational component that refers to the empathic and interpersonal spirit of MI, as well as a technical component that refers to the evocation and reinforcement of change talk [22]. In MI, open-ended questions, affirmation, reflective listening, and summarizing are referred to as core MI skills [23]. The method was first developed for treating alcohol abuse [23], and has later been shown to be effective in various clinical settings [24–27] and in brief interventions with small doses of 15 min [28] and single sessions [29]. While the evidence for MI as an effective RTW intervention is sparse [30–32], a Canadian cluster RCT found that the use of MI in rehabilitation led to more sustainable RTW compared to traditional rehabilitation for patients with musculoskeletal complaints [33, 34]. In a recent study we found that sick-listed workers receiving MI as an RTW intervention, given by caseworkers at NAV, experienced increased RTW self-efficacy [35]. A Swedish study on social insurance officers using MI in a sickness insurance setting found that the implementation of MI largely failed due to high workload and lack of support and priority from management [36].

Furthermore, MI quality has been found to vary between countries and cultures [37]. Thus, it is important to consider cultural and contextual differences when comparing measures of MI quality across studies and settings. When counseling the sick-listed workers in Norway, NAV recommend that their caseworkers use MI [37], but to what extent and quality is unknown. Thus, there is a lack of knowledge on how MI can be implemented as an RTW intervention [30]. The aim of this study was to evaluate potential barriers and facilitators for implementing MI as an RTW intervention in a Norwegian social insurance setting.

## Materials and Methods

This mixed-methods process evaluation was conducted alongside a RCT [20] and was guided by the RE-AIM framework [15, 19]. The project was carried out in cooperation with NAV and approved by the NAV management. The process evaluation components adoption, implementation and maintenance were chosen to evaluate potential barriers and facilitators for implementing MI as an RTW intervention. Reach and effectiveness of the intervention will be reported separately later on. Adoption, implementation and maintenance were assessed through focus groups with NAV caseworkers who administered the interventions, treatment fidelity through audio-recordings of MI sessions, and questionnaires to sick-listed workers who received the MI sessions. The manuscript is reported according to the Consolidated criteria for reporting qualitative research (COREQ) guideline.

### Study Setting: The Norwegian Welfare System and Sickness Absence Follow-Up

In Norway, employees are entitled to full wage benefits from the first day of sickness absence to a maximum period of 52 weeks. The employer is responsible for the payment during the first 16 working days, while the remaining period is covered by the National Insurance Scheme through NAV [39]. In terms of sickness absence follow-up, the employer and sick-listed worker share the main responsibility for all activities during sick leave and the RTW process. NAV has a facilitating role and is responsible for arranging a meeting within 26 weeks of sick leave, including the employer and the sick-listed worker. The attendance of the sick-listed employee’s general practitioner is optional, unless NAV deems it necessary for the continued coordination of the RTW process. Another meeting can be held if one or more of the stakeholders find it necessary. The sick-listed worker may also ask for a meeting at any time with NAV to coordinate a plan for RTW outside the scheduled follow-up [39]. Caseworkers at NAV working with the follow-up of sick listed workers have different educational backgrounds. A bachelor degree is required, but there are no requirements as to within what field. New caseworkers receive an introductory course about the regulations relevant for follow-up of sick-listed workers, resources available for providing work accommodation and job matching, as well as general information about different counselling techniques used within the social security setting. There is no mandatory training in counselling.

### The Interventions

The RCT was designed with three arms: the MI intervention, an active control group comprised of non-MI sessions, and a practice-as-usual group [21].

*The MI intervention* consisted of two MI counseling sessions that were offered to the sick-listed workers by a NAV caseworker (hereby after referred to as MI caseworker), in addition to the standard NAV follow-up. The first session was held at 14 weeks of sick leave and the second session at 16 weeks of sick leave, where each session had a maximum duration of 60 min. In the first session the emphasis was on engaging the sick-listed worker in forming a collaborative relationship with the caseworker, evoking the person’s own motivations to RTW, and mapping out an agenda for the session. During the first session, the caseworker also assessed the sick-listed worker’s readiness to change, their RTW self-efficacy and the current RTW plan if applicable. The NAV caseworkers also assessed the participant's level of RTW readiness according to the stages of change model [40], to adjust the intervention accordingly. In the second session the MI caseworker aimed to map the sick listed individual’s work tasks and earlier attempts of RTW. Information exchange of available support from NAV during the RTW process was included. The second session would ultimately result in one of two outcomes for the sick-listed worker: 1) The sick-listed worker was ready for change and/or ready to RTW and an action plan was written together with the NAV caseworker for establishing commitment to the plan. 2) If the sick-listed worker was not ready for change or RTW was not appropriate at this moment (e.g., lack of work adaptations, health issues), then no plan was written. Regardless of whether an RTW plan was made in the session, the caseworker provided a written summary of the two MI-sessions that was made available for the sick-listed worker at NAVs personal online service portal [21].

NAV had several years ago attempted to incorporate MI into the organization, and the nine caseworkers that were included in the present study had some MI experience from that period. The caseworkers volunteered to be selected for MI training in the present project. Before project start, these caseworkers were trained by two MI trainers (GB and RH), each with 15 years of MI training experience. One of the trainers (GB) is a member of Motivational Interviewing Network of Trainers. The MI intervention was carried out using a standardized MI guideline developed by the MI experts in our research group. The selected MI caseworkers had two three-hour workshops with basic MI training in early 2017. During the six months leading up to intervention start, all the MI caseworkers received three-hour training sessions every other week, which was later adjusted to every three weeks. When the intervention started, the MI training was comprised of three-hour sessions with supervision every third week, and from august 2018 it was reduced to three-hour sessions with supervision once a month. During the training, the caseworkers were guided to develop the necessary MI skills, in addition to receiving feedback from the MI experts on recorded MI sessions. Due to slow recruitment to the RCT, the number of caseworkers administering the MI intervention were reduced from nine to four. The four caseworkers who continued administering the MI intervention were those who wanted to continue. The other five caseworkers who had undergone the MI training acted as substitutes if needed (referred to as MI-substitute caseworkers) [21].

### Study Population and Recruitment

The study population consisted of caseworkers at NAV working with sickness absence follow-up who administered the MI intervention, and sick-listed workers who were enrolled into the RCT.

#### NAV Caseworkers

Eligible participants in the focus groups were MI caseworkers and MI-substitute caseworkers. E-mail addresses for all possible caseworkers in each group were forwarded by NAV management to the first author (VSF), who invited them to participate in the focus groups. All four of the possible caseworkers in the MI group agreed to participate and three out of five in the MI-substitute group agreed to participate in the focus groups. The NAV caseworkers had varying experience of working in NAV. In the MI group it ranged from 11 to 28 years, whereas for the MI-substitute group it ranged from 3.5 to 5.5 years. All the caseworkers who participated in the focus groups were female.

#### Sick-Listed Workers

Eligible participants for the RCT were sick-listed workers, 18–60 years old, living in central Norway, with unselected diagnoses and sick-listed for at least 8 weeks (with a current sick leave status of at least 50%). Exclusion criteria were pregnancy-related sick-leave and unemployment. The sick-listed workers were informed that participation in the study did not affect their rights to sickness benefits in any way.

### Process Measures

#### Adoption

Adoption refers to the willingness to initiate an intervention and how those administering the intervention react to it [15, 20]. By using focus groups, we explored whether the NAV caseworkers were willing to learn and use MI, and how they reacted to using MI during the RCT. A questionnaire was used to examine how sick-listed workers reacted to receiving the interventions, where participants were asked about satisfaction, usefulness and timing of the MI intervention.

#### Implementation

Implementation includes whether the elements, structures and resources are in place to adequately achieve a successful implementation and whether it was delivered as planned [15, 20]. This was assessed by focus groups to explore how the NAV caseworkers experienced the intervention in terms of whether resources and structures were adequately in place to administer the interventions as planned during the RCT. MI treatment fidelity was measured by using Motivational Interviewing Treatment Integrity (MITI) Coding Manual 4.2.1. [39].

#### Maintenance

Maintenance is the extent to which a program or policy becomes institutionalized or part of the routine organizational practices or policies [15, 20]. By using focus groups, we explored what structures and resources were in place for the NAV caseworkers to maintain the MI intervention and whether they would continue with the method after completion of the project.

### Data Collection

#### Focus Groups

Two focus groups were carried out in 2019 with the MI and MI-substitute caseworkers in order to investigate how they experienced learning and practicing MI, both in sessions that were part of the project and in their regular meetings with sick listed workers. The focus groups were based on a semi-structured interview guide with five main questions concerning: The caseworkers’ experiences with the MI sessions and the MI training, how the organization had adopted the intervention, how the caseworkers experienced performing extra follow-up sessions versus usual follow-up procedures and finally whether the caseworkers would continue to practice the MI method after the completion of the project. Both focus groups were asked about experienced barriers to and facilitators for the implementation of MI in daily practice. The focus groups lasted from 81 to 89 min and were audio-recorded and transcribed verbatim. Each focus group was held in a meeting room at the participants’ workplace and conducted by a moderator and co-moderator.

#### MI Fidelity

To assess the treatment fidelity, randomly selected audio-recordings of 20 MI sessions were collected from the four MI caseworkers on a voluntary basis during 2019. The recordings were transcribed and two MI experts in the research group selected a 20-min segment in each transcription for further coding. Transcripts were then sent to an external coding lab with objective raters [41] and coded according to the MITI Coding Manual 4.2.1. [32], which is well suited for measuring MI fidelity in various settings [43]. Global scores were reported on a five-point Likert scale, capturing the coders’ overall impression of how poorly or well the MI counselor met the dimensions measured, ranging from “Beginning proficiency” (low) to “Competency” (high). The threshold for “beginning proficiency” in the global technical score is ≥ 3, whereas for the global relational score the threshold is ≥ 4 [42].

#### Questionnaires

The MI caseworkers delivered questionnaires to the sick-listed workers who completed their second MI session between June 2018 and November 2019. The sick-listed workers were asked about their satisfaction with the MI sessions on a scale from 1 (very dissatisfied) to 5 (very satisfied), and how useful they felt the conversations were for them (scored on a scale from 1 (not useful) to 5 (very useful)). Participants were asked about the timing of the sessions with the questions: “Did the two follow-up conversations come at an appropriate timing for you?” (“yes” or “no” alternatives) and “If the conversations were to come at another time, when do you think it would have been best?” with six alternatives: “two months earlier,” “one month earlier,” “the timing was good,” “one month later,” “two months later,” or “three or four months later.”

### Data Analysis

The focus groups were analyzed using thematic analysis inspired by Braun and Clarke [44]. Thematic analysis is a flexible six-phased recursive process that allows the researcher to move back and forth between phases, which is suitable for analyzing focus group data [44]. Coding and theme development were according to the RE-AIM framework [15]. The analysis had five steps: 1) The authors read and reread the interviews to familiarize themselves and get an overall impression of the focus groups’ data and occurring patterns, where preliminary codes were identified. 2) Items of interest to the aim were coded and used to create core categories for the development of initial themes. 3) Codes were combined into initial themes and then 4) reviewed and checked against the coded data in order to expand or revise the developed themes. 5) All authors involved in the data analysis had several meetings to discuss and validate the final themes, as a part of the last step of the analysis, which is to define and refine the existing themes in order to tell a coherent and compelling story about the data [44]. Followingly, the thick descriptions were written into an analytical text [44], contextualized according to the RE-AIM framework [15]. The focus group participants read the final results in order to validate quotes and content. The analysis was first conducted on the data from the MI caseworker group, followed by deriving differences and nuances from the focus group with MI-substitute caseworkers.

Descriptive statistics were used to analyze the questionnaire and MITI data in Stata 15.1, College Station, TX [45].

### Ethics

The study was approved by the Regional Committee for Medical and Health Research Ethics in South East Norway (2016/2300). All focus group participants received written and oral information about the study and gave their written consent before the focus groups started. The sick-listed workers had given informed consent when being enrolled into the RCT study but provided specific consent for the questionnaire and audio-recordings of the 20 MI sessions.

## Results

The results from the two focus groups with NAV caseworkers, questionnaire data from sick-listed workers and MITI scoring from MI sessions are presented under the headings of the three RE-AIM components: adoption, implementation and maintenance.

In total, 180 sick-listed workers completed the two MI sessions during the period the questionnaires were distributed. A total of 112 (62.2%) participants responded, where 63% were women and the mean age was 45.5 years (SD 9.7). Sixty-two percent of the participants had higher education (Table 1). Participants included in the RCT during this period were mainly sick listed due to musculoskeletal complaints (38%) or mental health disorders (30%), the last third (32%) contained varied other diagnoses.

**Table 1:** Baseline characteristics of sick-listed workers answering the questionnaire about the MI intervention

	MI *N* = 112
*Gender*	
Female n (%)	70 (63%)
*Age*	
Mean (SD)	45 (9.7)
*Education level*	
High school n (%)	26 (23%)
College/university up to 3 years n (%)	44 (39%)
University more than 3 years n (%)	42 (38%)
*Sick leave length*^*a*^	
Less than 2 months n (%)	53 (48%)
2–4 months n (%)	38 (34%)
4–6 months n (%)	14 (13%)
6–8 months n (%)	3 (3%)
More than 8 months n (%)	3 (3%)

*MI intervention* motivational interviewing intervention

^a^Participants` length of sick leave at the time of the first MI session at NAV

### Adoption

Mastering various MI skills required considerable training and practice. However, using the MI method as an early follow-up intervention was experienced as meaningful for the MI caseworkers. Most sick-listed workers reported being satisfied with both the MI sessions and the timing of the MI sessions.

#### MI: A Useful But Difficult Method to Master

The MI training in the current project was experienced as good and comprehensive for the MI caseworkers, but the MI-substitute caseworkers emphasized the need for practicing MI in actual counseling sessions to further develop their skills. At the beginning of the project period, the MI caseworkers were uncertain about how to use the method and gave considerable effort to remembering which skills to use, and how and when to use them, rather than focusing on the content of the conversation. At this stage, they relied heavily on the MI guide developed by the research team. However, with time and practice, they were able to integrate the MI skills into their own conversation style, and retrospectively often realized that they had been using MI. Some specific MI skills were easier to master than others, such as asking open questions and reflective listening, since they were familiar to previous counseling approaches used prior to learning MI. Other MI skills, such as summarizing and asking for permission to give information or advice, were harder to incorporate and use, as it challenged their counseling habits and ability to stay focused throughout the MI session.

What I’m struggling the most with is summarizing, and I’m also struggling with staying focused, and then there’s also this ‘ask for permission’ stuff that I think is quite difficult. I think that it’s kind of unnatural to ask for permission to inform, since they come to us because they want to and then I assume they want to hear what we have to say, and I feel like “No I can’t, I should ask for permission to inform them,” which I take for granted, so I struggle a bit with that one [laughing]. – MI Caseworker 3.

Regardless of the difficulties, the MI caseworkers collectively expressed motivation to further develop their MI skills. The MI-substitute caseworkers described that they sporadically applied the MI method in their regular counseling. They experienced that their MI training helped them apply a more explorative approach in their regular counseling sessions by using MI skills such as summarizing and reflective listening.

The MI caseworkers reflected upon how, through MI, they had discovered a new way to meet the sick-listed workers’ needs, which enabled them to facilitate the sick-listed workers’ RTW process.

That is what we usually experience, that when sick-listed workers come to NAV they really want to talk about their health issues in order to be believed, and to get acceptance for their sick leave. Many people who get called into a meeting with NAV feel that there is an underlying distrust towards them, so they need to explain why they are on sick leave and describe their health problems. We just give them room to explain, and I think with MI, that if you use summarizing, it kind of helps me to show them that I have heard them, that I have respected them, and that what they are saying is true. It simply makes them feel understood, heard and trusted […]. – MI Caseworker 3.

The MI sessions were a meaningful experience for the MI caseworkers in several ways. First, it gave the caseworkers an opportunity to build a relationship with the sick-listed worker and explore their situation. Second, having two separate one-on-one counseling sessions at an early stage of sick leave gave the MI caseworker a chance to use their MI expertise to facilitate the sick-listed workers’ RTW process. The MI caseworkers preferred these early follow-up sessions as a way of delivering RTW follow-up, as opposed to the standard sickness absence follow-up at NAV, where they were are not involved before 26 weeks of sick leave. Third, these sessions were not only an opportunity to practice specific MI skills, but also a situation where they experienced professional growth and confidence in their abilities as caseworkers.

#### Satisfaction with the MI Sessions Among Sick-Listed Workers

The vast majority of sick-listed workers (99%) reported being satisfied or very satisfied with the MI sessions (Table 2). Most participants found the MI sessions to be useful (89%) and had an appropriate timing (86%), but at the same time 36% would have preferred the MI sessions to be either one or two months earlier than they were (Table 2).

**Table 2:** Frequencies of sick-listed workers’ satisfaction, usefulness and timing of the MI intervention

		1 Strongly disagree	2 Disagree	3 Neutral	4 Agree	5 Strongly agree
How satisfied were you with the conversations? n (%)	(n = 111)	1 (1%)	0	0	15 (13%)	95 (86%)
How useful was the first conversation for you? n (%)	(n = 112)	1 (1%)	4 (4%)	17 (15%)	36 (32%)	54 (48%)
How useful was the second conversation for you? n (%)	(n = 111)	2 (2%)	1 (1%)	11 (10%)	30 (27%)	67 (60%)
How useful were the conversations for you? n (%)	(n = 108)	1 (1%)	1 (1%)	10 (9%)	31 (29%)	65 (60%)

			Yes	No			
Did the two follow-up conversations come at an appropriate timing for you?	(n = 112)		96 (86%)	16 (14%)			
		2 months earlier	1 month earlier	The timing was good	1 month later	2 months later	3 or 4 months later
If the conversations were to come at another time, when do you think it would have been best? (n = 99)		21 (21%)	15 (15%)	61 (62%)	1 (1%)	1 (1%)	0

### Implementation

The MI caseworkers experienced the MI interventions as being an extra workload, while also experiencing a lack of support and follow-up from the NAV management. Furthermore, conflicting roles when counseling sick-listed workers were experienced as a problem for the MI caseworkers.

#### Resource-Intensive RTW Follow-Up

The MI sessions were described as a strain by the caseworkers, as this added two extra 60-min sessions to their usual schedule of sickness absence follow-up. The MI sessions also required time for preparation in order to perform at an appropriate level. For the caseworkers delivering MI, it was challenging to move from the usually goal-oriented counseling setting, to counseling sessions where the process was a goal in itself. However, the exploratory style of MI allowed caseworkers to better understand the complexity of the sick-listed workers’ situation, which enabled better tailoring of the sick-listed workers’ RTW process.

The MI sessions feel very different than those we have in the usual follow-up context, where they perhaps are more planned and goal-oriented. We are more focused on getting somewhere in the usual follow-up sessions than in the MI sessions, where we don’t have this focus. I have more of an exploratory focus because of the way the conversations [MI sessions] are organized, with the agenda of exploring all areas of people’s lives. I notice that, yes, I spend a lot more time doing these sessions and that I have to limit myself more than I usually would. […]. It is really different from what I am used to doing in regular meetings. – MI Caseworker 2.

Based on communication with NAV management prior to the project, the MI caseworkers had assumed that they would have a reduction in their regular caseload in order to compensate for the added workload. However, the MI caseworkers did not experience any reduction in their ordinary workload. Conflicting roles when counseling the sick-listed workers was also experienced as problematic for the MI caseworkers.

We felt that we should be in the MI-spirit in order to explore the sick-listed workers’ motivation to move forward, while also being gatekeepers of sickness benefits […]. We are supposed to wear many hats, but it can be challenging when it comes to the expectations of me as a NAV caseworker in an MI context. I am in fact a NAV caseworker, who is supposed to provide information about rights and obligations for sickness benefits. – MI Caseworker 1.

On the one hand they were supposed to help individuals and develop a good relationship with the sick-listed workers, while on the other hand they were gatekeepers to sickness benefits. Using MI was more indicative of the first role than the latter, and they frequently described removing the “MI hat” in order to talk about rules, laws, rights and obligations.

#### MI Fidelity

The MI fidelity results assessed by MITI showed technical global scores over the threshold for “beginning proficiency” with an average of 3.26 (SD 0.69), whereas the relational global score was under the threshold for “beginning proficiency”, with an average of 3.45 (SD 0.63) (Table 3).

**Table 3:** MI treatment fidelity scores of MI caseworkers

MITI components	Mean (SD)
*Global ratings*	
Technical: cultivating change talk	3.11 (0.94)
Technical: softening sustain talk	3.34 (0.67)
Relational: partnership	3.55 (0.67)
Relational: empathy	3.35 (0.73)
Global score technical	3.26 (0.69)
Global score relational	3.45 (0.63)

*MITI* motivational interviewing treatment integrity, *SD* Standard deviation

### Maintenance

Maintaining MI skills was difficult for the MI caseworkers, as they had no appropriate or organized place for utilizing and practicing their MI skills outside of the MI sessions and the monthly supervisions.

#### Maintaining MI Skills: The Need for Practicing

The MI caseworkers had doubts about their MI skills and expressed the need for practical experience in order to recognize and learn when they were using MI. Thus, they emphasized the need for continuous practice if this intervention was to be maintained in regular follow-ups.

Now that we have begun to learn the ropes of it, some of the things we struggled with in the beginning have begun to go on autopilot, but there is still some insecurity amongst us sometimes. […]. We have all had this kind of insecurity and we often get confirmation in our MI training that we aren’t as bad [at MI] as we might think. – MI Caseworker 2.

Although they experienced that their MI training enabled them to be more explorative when counseling sick-listed workers, they experienced a lack of competence in MI skills. This led to stressful MI sessions, making it difficult for them to focus on the task at hand. In order to cope with the stress, discussing and sharing MI experiences with colleagues was considered important. However, based on earlier attempts at incorporating MI into the organization some years ago, the MI caseworkers described resistance towards the MI approach among several of their co-workers. This made it hard to receive the needed support in order to maintain and further develop their MI skills. The MI caseworkers discussed whether the MI sessions were the only suitable place for training in the MI method in NAV, as regular follow-up sessions involved other stakeholders, and that MI was considered best suited for one-on-one sessions.

## Discussion

The aim of this study was to evaluate potential barriers and facilitators for implementing MI as an RTW intervention in a Norwegian social insurance setting. The main barrier for adopting MI was the amount of time needed for training and practice in order to master the various MI skills. The barriers to implementing MI were that delivering the MI intervention was experienced as resource-demanding and an extra workload, in addition to a lack of co-worker and managerial support. The main barriers for maintenance was that MI caseworkers had no appropriate time and place for practicing the further development of MI skills. MITI scores showed that the MI caseworkers were over the threshold for “beginning proficiency” in the technical component of MI, whereas they scored beneath the threshold in the relational component. The main facilitators for implementing MI were the motivation to learn and master the MI method. Furthermore, early follow-up sessions allowed the caseworkers to understand the complexity of the sick-listed workers’ life situation and believed they could positively influence the RTW process. Regarding the sick-listed workers, they reported that they were satisfied with the MI sessions.

Evoking motivation and change, which are essential parts of MI [22], were experienced as difficult skills to master for all the caseworkers who underwent MI training in the current study. Despite thorough training and mentoring, the MITI scores showed that the technical component of MI was at a “beginning proficiency,” while the relational component was beneath this threshold. A possible explanation for the low relational MITI score might be that the caseworkers were too much focused on the technical side when using MI. The low relational MITI scores are contradicted by the results from the interviews of the sick-listed workers where they experienced the MI-caseworkers as being both empathic and showing genuine interest in their situation [35]. These low MITI scores are in line with a previous study that found that neither workshops nor additional supervision were sufficient for reaching “beginning proficiency” levels in MI [46]. Another recent study, however, found that four hours of MI training significantly increased counselors’ MI competence scores, as well as their skills to promote clients’ engagement in RTW behaviors and a strong working alliance [47]. MI providers’ level of skills is known to vary within and between providers [48], and skill levels are suggested to be related to treatment outcome, even though the results are inconsistent [49]. However, it is unknown what level of technical MI proficiency is needed to affect RTW outcome. It should also be noted that in a recent study we found that the sick-listed workers reported that they experienced the MI sessions as enabling in terms of RTW strategies [35].

The low MITI scores in the present study could suggest that barriers such as extra workload, lack of support and practicing can negatively affect maintaining MI performance in a social insurance setting, despite extensive initial training. This is in line with previous studies, where the implementation of MI largely failed due to high workload and lack of managerial support and priority [36], and where reduced workload and higher flexibility were considered facilitating factors when using MI [50]. Developing high levels of MI competence for caseworkers in a social insurance setting can be a difficult endeavor due to these barriers. The current project was externally funded and therefore better resourced in terms of training, supervising and administering MI than in regular NAV practice. A lack of such resources in ordinary social insurance settings could hinder achieving desired proficiency of MI skills. Similarly, a lack of MI proficiency might also hinder MIs efficacy on RTW, thereby suggesting that implementation of MI is not realistic without sufficient resources in place.

NAV caseworkers operate both as supporting RTW professionals and controllers of sickness benefits [38]. These conflicting roles represents a double role paradox that could contribute to ambivalence in decision-making for the caseworkers [51] and hinder the establishment of a good relationship when helping individuals to RTW [52]. This illustrates that the adaptation of MI in a non-therapeutic social insurance setting may be hindered by conflicting roles and the amount of information exchange necessary in a counseling session at NAV. In the present study, the MI caseworkers described that using MI was more indicative of being an RTW professional rather than a gatekeeper for sickness benefits. Putting on the proverbial “MI hat” helped the caseworkers to focus on establishing a good relationship, rather than focusing on entitlement to sickness benefits. Thus, MI may be beneficial as a tool in reducing the barrier that the double role paradox poses on the sick-listed workers and caseworkers’ cooperation in the RTW process. In addition to the caseworkers’ motivation to learn and master MI, this may be considered an important facilitating factor for implementing MI in a social insurance setting, as this may enable the caseworkers to positively influence the sick-listed workers’ RTW process.

It has previously been reported that sick-listed workers experience the content and timing of standard follow-up given by NAV as being insufficient to facilitate RTW [53]. In the present study, meeting sick-listed workers at an earlier stage of sick leave was experienced as a preferred way of conducting sickness absence follow-up. In addition, the sick-listed workers were satisfied with the timing of the MI intervention. This suggests that there is a call for more early one-on-one counseling meetings in sickness absence follow-up in Norway.

## Strengths and Limitations

A strength of the current study was the use of mixed-methods, using NAV caseworker focus groups, MI fidelity scores and questionnaires for sick-listed workers to explore the adoption, implementation and maintenance of the interventions using the RE-AIM framework [15]. The findings are validated through the contribution of our interdisciplinary group of researchers throughout the analytical process. Since the RCT is ongoing, the reach and effectiveness of the intervention will be reported separately later on. Consequently, the current process evaluation was not biased by effectiveness measures when evaluating the implementation of MI. All the caseworkers in this study were women. Women represent the majority of social insurance officers in Norway.

Since the current project was externally funded it was better resourced in terms of training, supervising and administering MI than regular NAV practice. However, the volume of MI sessions would be higher if this was regular practice, and not limited by slow recruitment, as was the case in the current RCT. Another limitation is the lack of data on the managerial perspective, which could have balanced the caseworkers’ experiences about workload and added information on structural and organizational barriers and facilitators for the implementation of MI in NAV. Furthermore, as this study was a collaboration with NAV, the caseworkers may have felt obligated to participate in the focus group study, and it could have affected what they chose to share. Furthermore, the selection of the MI caseworker group through their motivation for learning and using MI may have biased the caseworkers to overestimate the advantage of MI in practice. Background information regarding the caseworkers’ education and experiences with previous or other counselling techniques would have strengthened the study. Furthermore, recording MI fidelity on a voluntary basis during a limited period of time in the early phase of the RCT may have affected the representativeness of the MI fidelity scores.

## Conclusion

Adopting and implementing MI as an RTW intervention in a social insurance setting required significant resources. The MI method was hard to master and use in practice, a notion that was supported by the low MITI scores. However, more research is needed on how technical and relational MI skills affect RTW outcomes. A lack of co-worker and managerial support, time and place for practicing and developing MI skills, and a high workload were identified as barriers to implementing MI. More attention should be paid to reducing these barriers and to investigate whether this would promote implementation of MI in a social insurance setting. Similarly, promoting facilitating factors, such as caseworker motivation and early follow-up sessions, may be equally important for implementing MI in a social insurance setting. However, there is an urgent need for well-designed effect studies of MI on RTW to justify the investments required to adopt, implement and maintain MI as a main tool in early follow-up of workers on sickness absence.

## Data Availability

The data sets generated and analyzed during the current study are not publicly available due to protecting the anonymity of participants, but redacted versions are available from the corresponding author on reasonable request.

## Abbreviations

MI: Motivational interviewing
MITI: Motivational interviewing treatment integrity
NAV: Norwegian Labour and Welfare Administration
RCT: Randomized controlled trial
RE-AIM: Reach, effectiveness, adoption, implementation, and maintenance
RTW: Return to work
